# Relationship Between ABO/Rh Blood Groups and Knee Osteoarthritis: A Retrospective Cohort Study

**DOI:** 10.3390/jcm15072656

**Published:** 2026-03-31

**Authors:** Gurkan Gumussuyu, Kaan Saritas, Belkis Koctekin, Serkan Gurcan, Ozkan Kose

**Affiliations:** 1 Vocational School of Health Services, Istinye University, 34010 İstanbul, Turkey; gurkangum@yahoo.com; 2 Department of Orthopedics and Traumatology, Antalya Education and Research Hospital, University of Health Sciences, 07100 Antalya, Turkey; kaansaritas@gmail.com; 3 Transfusion Center, Antalya Training and Research Hospital, University of Health Sciences, 07100 Antalya, Turkey; bkoctekin@gmail.com; 4 Private Practice, Gurcan Orthopedics Clinic, Cevizlik, 34365 İstanbul, Turkey; serkangurcan@yahoo.com

**Keywords:** osteoarthritis knee, arthroplasty replacement knee, ABO Blood-Group System, Rh-Hr Blood-Group System, blood group antigens

## Abstract

**Background/Objectives**: The association between ABO/Rh blood groups and knee osteoarthritis (OA) remains controversial, with inconsistent findings reported across different populations. This study aimed to evaluate the distribution of ABO and Rh(D) blood groups in patients undergoing primary total knee arthroplasty (TKA) for primary knee OA and to compare these distributions with a regional external reference population. **Methods**: This retrospective, single-center, observational study reviewed hospital records of patients who underwent primary TKA between January 2011 and October 2024. After applying predefined exclusion criteria (different ethnic background, age < 50 years, secondary knee OA, and missing blood group data), 4969 patients with primary knee OA were included. ABO/Rh(D) data were obtained from the institutional electronic hospital information system and transfusion/laboratory records. The external reference population consisted of a previously published dataset of regional blood donors (10,867 unique donors). Observed blood group frequencies in the study cohort were compared with expected frequencies derived from the reference distribution using chi-square goodness-of-fit tests for (1) 8-category ABO/Rh(D) distribution, (2) ABO-only distribution, and (3) Rh(D)-only distribution. **Results**: Among 4969 patients, 4096 (82.4%) were female and 873 (17.6%) were male. Mean age was 66.8 ± 7.0 years (range, 50–94) in females and 68.8 ± 7.3 years (range, 50–88) in males. The most frequent blood groups were A Rh (+) (39.3%), O Rh (+) (30.0%), and B Rh (+) (14.5%). The sex-specific ABO/Rh distribution did not differ significantly (*p* = 0.052). Compared with the regional reference distribution, the overall 8-category ABO/Rh(D) distribution showed a borderline difference (χ^2^ (7) = 14.04, *p* = 0.050; Cramér’s V = 0.020). However, neither the ABO-only distribution (χ^2^ (3) = 5.26, *p* = 0.153; Cramér’s V = 0.019) nor the Rh(D)-only distribution (χ^2^ (1) = 0.11, *p* = 0.737; Cramér’s V = 0.005) differed significantly from the regional reference. The observed deviations were numerically small and not suggestive of a clinically meaningful difference. **Conclusions**: In this large single-center cohort of patients undergoing primary TKA for primary knee OA, the ABO and Rh(D) blood group distributions were largely comparable to those of the regional population. Although the overall 8-category ABO/Rh(D) comparison showed a borderline difference, separate ABO-only and Rh(D)-only analyses were not significant. These findings do not support a strong association between blood group status and surgically treated primary knee OA in this population.

## 1. Introduction

Knee osteoarthritis (OA) is a chronic degenerative joint disease characterized by progressive articular cartilage degeneration, subchondral bone remodeling, and gradual loss of joint function [1]. Its prevalence continues to increase worldwide, particularly in aging populations, making it one of the most important causes of pain, disability, and impaired quality of life in older adults [2]. In advanced stages, knee OA frequently leads to total knee arthroplasty, thereby creating a substantial surgical and economic burden on healthcare systems [3]. Although OA has traditionally been viewed as a “wear-and-tear” disorder, contemporary evidence has demonstrated that it is a complex, multifactorial disease in which inflammatory pathways play a substantial role in both disease initiation and progression. In particular, pro-inflammatory mediators such as tumor necrosis factor-α (TNF-α) and interleukins contribute to cartilage degradation by modulating the biological interplay among the synovial membrane, articular cartilage, and subchondral bone [4,5,6,7,8].

In recent years, the ABO blood group system, a genetically determined biological marker, has attracted increasing attention due to its potential associations with systemic inflammatory responses and susceptibility to various diseases, including cardiovascular disease, diabetes mellitus, allergic diseases, gastrointestinal diseases, and cancer [9,10,11,12,13,14,15,16,17]. ABO antigens are expressed not only on erythrocytes but also on a variety of non-hematologic cells and tissues, including epithelial and endothelial cells; they have also been reported in joint-associated musculoskeletal tissues, such as synovial tissue [18,19]. Several mechanisms have been proposed to explain a possible relationship between blood group antigens and OA. These include associations of the ABO gene locus with circulating inflammatory mediators (notably sICAM-1, and in some reports TNF-α), as well as potential local effects of blood group-related antigens expressed in joint tissues (including synovial tissue and the pro-inflammatory Lewis Y [LeY] antigen) on cartilage metabolism and joint homeostasis [19,20,21,22].

Despite these biologically plausible mechanisms, the literature investigating the relationship between ABO blood groups and knee OA remains limited, and the available findings are inconsistent [19,23,24,25,26]. Li et al. have suggested that blood group AB may be an independent risk factor for knee OA [19]. In contrast, studies from Pakistan [24] and Türkiye [25] have reported a stronger association between blood group A and the presence of knee OA and/or revision-related outcomes. On the other hand, a report from Nigeria, representing a different geographic and ethnic population, failed to demonstrate a significant association between blood group status and disease severity [26]. These discrepancies may reflect differences in study design, sample size, outcome definitions, disease stage, and population-level distributions of blood groups across ethnicities or regions. Therefore, the current evidence base remains inconclusive, and an important gap persists in the literature. Accordingly, the present study was designed as a distributional comparison of ABO and Rh(D) blood groups in patients undergoing primary total knee arthroplasty for advanced primary knee OA, using a regional external reference population, rather than as a direct assessment of OA susceptibility or genetic predisposition.

Based on these considerations, we hypothesized that ABO and Rh(D) blood group distributions in patients undergoing primary TKA for advanced knee OA might differ from those of the regional reference population. Accordingly, the aim of the present study was to evaluate the distribution of ABO and Rh(D) blood groups in a large cohort of patients who underwent primary total knee arthroplasty for knee OA and to compare these distributions with those of a regional external reference population from the same geographic setting.

## 2. Materials and Methods

### 2.1. Patients and Study Design

This study was designed as a retrospective observational study conducted at a single tertiary referral center in Antalya, Türkiye. Hospital records were reviewed to identify all patients who underwent primary total knee arthroplasty (TKA) between January 2011 and October 2024. Demographic characteristics and ABO/Rh blood group information were extracted from the institutional electronic hospital information system and transfusion/laboratory records.

**Figure 1:**
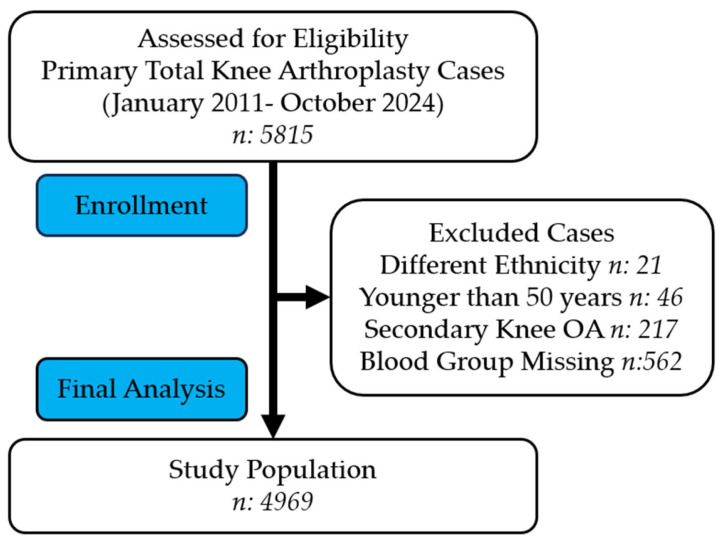
The flow diagram of patient selection.

A total of 5815 primary TKA cases were initially screened. Patients were then excluded according to the predefined criteria shown in the study flow chart (Figure 1). After exclusions, 4969 patients with primary knee osteoarthritis constituted the final study population. Since this was a retrospective record-based study, only patients with complete blood group records were eligible for the final analysis.

The primary aim of the study was to evaluate whether the ABO/Rh blood group distribution among patients undergoing primary TKA for knee osteoarthritis differed from that of the external regional reference population (Antalya blood donor distribution). Because the study question focused on blood group distribution in surgically treated primary knee osteoarthritis, patients with secondary OA etiologies (such as post-traumatic knee OA, inflammatory arthropathies) were excluded to reduce etiologic heterogeneity and confounding. Patients with different ethnicities were also excluded, as ethnic variation may affect ABO/Rh blood group frequencies and thereby compromise the validity of comparisons with the regional Antalya reference distribution. Ethnicity was inferred from patients’ names/surnames and place of birth, as recorded in the medical records. Furthermore, despite the chart review, patients younger than 50 years were excluded to minimize potential misclassification. This age threshold was selected as a pragmatic eligibility criterion in this retrospective study because secondary causes of knee osteoarthritis, particularly post-traumatic degeneration, are relatively more common in younger individuals than in patients with typical age-related primary OA. Therefore, restricting the cohort to patients aged 50 years or older was intended to increase the likelihood that the final sample would predominantly represent primary knee OA rather than secondary forms of the disease [27,28]. Patients with missing ABO/Rh data were excluded because blood group status was the primary exposure variable.

Approval from the University of Health Sciences, Antalya Education and Research Hospital Local Ethics Committee was obtained prior to the study (Approval date: 5 February 2026, No: 3/14). The study was conducted in accordance with the Declaration of Helsinki and its later amendments. Patient consent was waived due to the retrospective, file-based design of the study.

### 2.2. External Reference Population for Turkish ABO/Rh Distribution

To contextualize the observed ABO/Rh frequencies in our cohort, a regional Antalya reference distribution was used as an external reference population [29]. Reference proportions were adopted from a retrospective donor-based study conducted at the Antalya Training and Research Hospital Transfusion Center, which evaluated ABO and Rh blood group distributions among blood donors who applied between 1 January 2013 and 1 September 2019. In that study, 15,997 donor applications were screened, and 10,867 unique donors were included after duplicates were identified through national identification records, thereby minimizing repeated-measures bias. Blood groups were determined using a microcolumn gel agglutination method in the immunohematology laboratory. The reported overall distribution in the Antalya donor population is presented in Table 1. This regional reference was preferred because it more accurately reflects the local population structure of the study setting (Antalya, Türkiye) and reduces potential bias due to geographic variation in blood group distribution.

### 2.3. ABO/Rh Determination and Data Collection

ABO and Rh(D) blood group data were retrospectively obtained from the institutional electronic hospital information system and transfusion/laboratory records. Age, sex, and blood group data were recorded for all eligible patients included in the final analysis. At our institution, ABO/Rh typing is performed in the transfusion center immunohematology laboratory as part of routine preoperative evaluation using a standardized institutional workflow. Blood group determination is based on the hemagglutination principle with a microcolumn gel centrifugation method. In the center’s routine practice, gel centrifugation cards used for ABO/Rh typing include the Across Gel (Dia Pro Medical Products, Istanbul, Türkiye). This is consistent with the methodology reported in the Antalya regional donor reference study conducted at the same center. Because both the study cohort and the external Antalya reference dataset originated from the same institution, blood group determination was considered methodologically comparable at the laboratory level. For analysis, patients were categorized into eight ABO/Rh groups [A Rh (+), A Rh (−), O Rh (+), O Rh (−), B Rh (+), B Rh (−), AB Rh (+), and AB Rh (−)] and additionally into ABO-only (A, O, B, AB) and Rh-only [Rh (+)/Rh (−)] groups.

### 2.4. Statistical Analysis

All analyses were carried out using IBM SPSS Statistics for Windows, version 23.0 (IBM Corp., Armonk, NY, USA). Statistical analyses were performed to compare the observed ABO/Rh(D) distribution in the study cohort with the expected distribution derived from the external Antalya regional reference population. Continuous variables were summarized as mean ± standard deviation (SD) and range. Categorical variables were presented as numbers (*n*), percentages (%), and 95% confidence intervals (CIs), where applicable. The primary analysis compared the 8-category ABO/Rh distribution [A Rh (+), A Rh (−), O Rh (+), O Rh (−), B Rh (+), B Rh (−), AB Rh (+), AB Rh (−)] of the study cohort with the Antalya reference distribution using the chi-square goodness-of-fit test. Expected counts for each category were calculated by multiplying the Antalya reference proportions by the total sample size of the study cohort. Secondary analyses were performed for ABO-only distribution (A, O, B, AB) and Rh(D)-only distribution [Rh (+), Rh (−)], again using chi-square goodness-of-fit tests against the corresponding Antalya reference proportions. To explore which categories contributed most to the overall chi-square statistic, observed and expected counts were compared, and standardized residuals were examined. Effect size was assessed using Cramér’s V. Because three related goodness-of-fit analyses were performed (8-category ABO/Rh(D), ABO-only, and Rh-only), the overall findings were also interpreted with consideration of multiple testing; under a Bonferroni-adjusted threshold (*p* < 0.0167), the borderline 8-category result would not be considered statistically significant. A two-sided *p*-value < 0.05 was considered statistically significant. Because the external reference population was based on an independent regional donor dataset, no matching procedure was performed; therefore, the results were interpreted as a distributional comparison rather than a causal risk analysis. Accordingly, odds ratios, relative risks, and multivariable-adjusted estimates could not be derived within the framework of the present study design.

## 3. Results

A total of 4969 patients were included in the final analysis. Of these, 873 (17.6%) were male, and 4096 (82.4%) were female. The mean age of female patients was 66.8 ± 7.0 years (range, 50–94), whereas the mean age of male patients was 68.8 ± 7.3 years (range, 50–88). The overall ABO/Rh blood group distribution of the study cohort, along with its sex-specific breakdown, is presented in Table 2. Among all patients, the most common blood group was A Rh (+) (*n* = 1952, 39.3%), followed by O Rh (+) (*n* = 1492, 30.0%) and B Rh (+) (*n* = 721, 14.5%). The distribution of ABO/Rh blood groups showed no statistically significant difference between females and males (*p* = 0.052), although this result was near the conventional significance threshold.

When the observed blood group distribution in the study cohort was compared with the expected distribution derived from the Antalya regional donor reference population, the overall 8-category ABO/Rh(D) distribution showed a borderline difference (chi-square goodness-of-fit test: χ^2^ (7) = 14.04, *p* = 0.050; Cramér’s V = 0.020), indicating a very small effect size (Table 3). Evaluation of observed versus expected counts in the 8-category analysis showed that the largest deviations were seen in AB Rh (+) (higher than expected; standardized residual = +2.33), AB Rh (−) (lower than expected; standardized residual = −1.80), and O Rh (+) (lower than expected; standardized residual = −1.77). However, these deviations were numerically small and do not indicate a clinically meaningful shift in the overall distribution relative to the Antalya regional reference population. Moreover, because three related goodness-of-fit analyses were performed, this borderline result would not remain statistically significant under Bonferroni correction (adjusted significance threshold: *p* < 0.0167).

In the secondary analyses, the ABO-only distribution (A, O, B, AB) did not differ significantly from the Antalya reference distribution (χ^2^ (3) = 5.26, *p* = 0.153; Cramér’s V = 0.019) (Table 4). Similarly, the Rh(D)-only distribution [Rh (+), Rh (−)] was also not significantly different from the regional reference (χ^2^ (1) = 0.11, *p* = 0.737; Cramér’s V = 0.005) (Table 5). These effect sizes were also negligible.

## 4. Discussion

In this large single-center retrospective cohort of patients undergoing TKA for primary knee OA, the principal finding was that the ABO and Rh(D) blood group distributions were largely comparable to those of the regional Antalya reference population. Although the overall 8-category ABO/Rh(D) comparison showed a borderline difference, neither the ABO-only nor the Rh(D)-only distribution differed significantly from the external regional reference population. In addition, the effect size for the 8-category comparison was very small (Cramér’s V = 0.020), and the observed deviations were numerically limited, indicating that the borderline *p*-value should not be interpreted as evidence of a clinically meaningful difference. These findings do not support a strong association between blood group status and surgically treated primary knee OA in our population. Importantly, this study did not evaluate OA incidence or population-level susceptibility; instead, it compared blood group distributions in a selected subgroup of patients with advanced primary knee OA undergoing surgical treatment.

A borderline difference was observed in the sex-specific ABO/Rh distribution within the surgical cohort. However, because this result was not statistically significant, it should not be interpreted as evidence of a true sex-specific biological association. Given the marked female predominance of the cohort and the smaller male subgroup, this finding is more likely attributable to sample imbalance than to a reproducible biological effect. Therefore, it should be regarded as exploratory and interpreted with caution.

Compared with previous studies, the current findings contribute to a heterogeneous body of evidence on the relationship between ABO blood groups and osteoarthritis. As summarized in Table 6, earlier studies have reported inconsistent associations across different populations and OA phenotypes. The Chinese case–control study by Li et al. reported blood group AB as an independent risk factor for primary knee OA and also linked synovial LeY antigen expression with disease severity [19]. Studies from Türkiye [25] and Pakistan [24] suggested a stronger association with blood group A (particularly A+) in knee OA cohorts. In contrast, the Nigerian study did not demonstrate a significant association between ABO blood group and severe knee OA, and the older hip OA study by Lourie reported only a modest pattern difference, with a non-significant overall ABO comparison but a lower frequency of group O in OA patients [23,26]. These collectively indicate that the direction and magnitude of association may vary across settings and study designs. Accordingly, our findings should be interpreted as reflecting the blood group distribution in end-stage surgically treated primary knee OA rather than as direct evidence of disease susceptibility in the general population.

Our results are more closely aligned with the studies reporting no clear or only weak association, particularly when the comparison is performed against a geographically appropriate reference distribution. Unlike several prior reports that suggested a dominant effect for a specific ABO group (e.g., AB or A), we found only a borderline difference in the overall 8-category ABO/Rh(D) distribution and no significant differences in the ABO-only or Rh(D)-only analyses. A key methodological distinction of the present study is the use of a regional external reference population from the same institution in Antalya, which likely reduced bias due to geographic variation in blood group frequencies and inter-laboratory differences in blood typing methods. This may partly explain why our findings were more conservative than studies using non-local or less comparable control populations.

Several factors may explain the between-study heterogeneity observed in the current literature. First, population-level differences in ABO/Rh distribution across geographic and ethnic groups may substantially influence apparent associations when external controls are not regionally matched. Second, studies differ in case definition and phenotype selection, including primary knee OA, severe OA, revision arthroplasty cohorts, or even hip OA, which are not necessarily biologically equivalent and may reflect different pathophysiologic pathways. Third, control selection strategies vary considerably (hospital-based controls, donor populations, regional/national reference datasets, or no formal control arm), which may introduce selection bias and limit cross-study comparability. In addition, differences in sample size, statistical modeling, and the specific outcome tested (overall ABO distribution vs. a single blood group effect; susceptibility vs. severity vs. revision risk) may contribute to inconsistent findings. In the present study, we attempted to minimize these sources of heterogeneity by restricting the cohort to surgically treated primary knee OA, excluding likely secondary OA cases, and using a geographically matched Antalya reference distribution from the same institutional setting. Moreover, to the best of our knowledge, this study includes one of the largest patient cohorts evaluated for this specific research question. However, this numerical strength should be interpreted in the context of the study’s retrospective design and distribution-based analytical framework.

A biologically plausible link between the ABO blood group system and knee osteoarthritis has been proposed primarily through inflammatory and glycosylation-related pathways. ABO antigens are not restricted to erythrocytes; they are also expressed on epithelial and endothelial surfaces and in other tissues, suggesting that ABO-related glycosyltransferase activity may influence disease processes beyond transfusion biology [30,31]. These glycosylation differences may alter cell-surface carbohydrate patterns, thereby influencing leukocyte adhesion, endothelial interactions, and the regulation of pro-inflammatory mediators within the synovial microenvironment. Li et al. specifically emphasized that OA pathogenesis involves inflammatory crosstalk among synovium, cartilage, and subchondral bone, and proposed that ABO-related variation may modulate this milieu by altering pro-inflammatory mediators [19]. In addition, prior genetic and epidemiologic studies have shown that the ABO locus is associated with several systemic disease phenotypes and inflammatory/vascular traits, supporting the concept that ABO status may reflect broader biologic differences rather than a purely hematologic characteristic [22,32]. A more OA-specific mechanistic hypothesis was raised by Li et al., who reported an association between LeY antigen expression in synovial tissue and primary knee OA, suggesting that blood group–related carbohydrate antigens may have local effects on synovial inflammation and cartilage metabolism [19]. However, these mechanisms remain hypothesis-generating, and current clinical association studies (including the present study) do not yet establish causality; thus, any ABO–OA relationship is likely to be modest and potentially modified by population structure, phenotype definition, and other host factors [19,26]. These proposed pathways remain speculative in the context of the present study, because no inflammatory biomarkers, genetic variants, or tissue-level antigen expression data were evaluated; therefore, our findings cannot directly confirm or refute these biological mechanisms.

The present study has several strengths. First, it included a large surgical cohort of 4969 patients with primary knee OA undergoing primary TKA, which may improve the precision of the distributional analysis compared with many previous reports in this field. Second, the study used a geographically matched external reference population rather than a nationwide distribution, which may help reduce bias related to regional variation in ABO/Rh frequencies. Third, both the patient cohort and the regional reference data were derived from the same institution, and blood group determination was performed within the same transfusion/immunohematology setting, supporting laboratory-level methodological comparability. Finally, the analysis was performed at multiple levels (8 ABO/Rh(D) subtypes, ABO-only, and Rh(D)-only), allowing for a more detailed assessment of whether any observed pattern was driven by ABO subtype composition or Rh status alone.

This study also has important limitations. First, its retrospective single-center design may introduce selection bias and limit generalizability to other populations. Second, although a regional control distribution was used, the external reference was still based on a blood donor population, which may not fully represent the general community because donor eligibility criteria and sex distribution can differ from those of patients undergoing TKA; therefore, this comparator should be regarded as a pragmatic regional reference rather than an ideal population-matched control cohort. In addition, because our surgical OA cohort was predominantly older and female, whereas blood donors are generally healthier and often younger and male, some degree of demographic and biologic selection bias cannot be excluded. Third, the analysis compared group distributions and therefore should not be interpreted as a causal risk analysis; no matching or multivariable modeling was performed against an individual-level control cohort. In addition, because the external donor-based reference dataset was available only as an overall regional distribution rather than an age- and sex-stratified individual-level comparator, more detailed subgroup analyses against the reference population were not performed. In addition, potentially relevant covariates such as body mass index, comorbidities, radiographic severity, inflammatory biomarkers, and genetic background were not available for adjustment in the present design. Ethnic background was inferred from names/surnames and place of birth recorded in the medical records rather than from self-reported ethnicity; therefore, some degree of misclassification cannot be excluded. Finally, because the cohort consisted of patients with surgically treated advanced primary knee OA, the findings may not be directly applicable to patients with earlier-stage disease or non-surgical OA populations.

## 5. Conclusions

In conclusion, in this large single-center cohort of patients undergoing primary total knee arthroplasty for primary knee osteoarthritis, the ABO and Rh(D) blood group distributions were largely comparable to those of the regional Antalya reference population. Although the overall 8-category ABO/Rh(D) comparison showed a borderline difference, ABO-only and Rh(D)-only analyses were not statistically significant. These findings do not support a strong association between blood group status and surgically treated primary knee OA in our population. Further multicenter, individually matched, and methodologically harmonized studies are needed to determine whether ABO/Rh status shows any reproducible association across different osteoarthritis phenotypes, stages, and clinical populations.

## Figures and Tables

**Table 1:** Regional ABO and Rh(D) distribution in Antalya, Türkiye (blood donors, 2013–2019). (Adapted from the Antalya donor-based blood group distribution study [29].

Blood Group	*n*	%	ABO Total (%)
A Rh (+)	4239	39.00	43.02
A Rh (−)	437	4.02	
O Rh (+)	3415	31.43	34.88
O Rh (−)	375	3.45	
B Rh (+)	1556	14.32	15.60
B Rh (−)	139	1.28	
AB Rh (+)	620	5.71	6.50
AB Rh (−)	86	0.79	
Total	10,867	100	100

**Table 2:** Distribution of ABO/Rh blood groups in the study cohort and according to sex.

Blood Group	Total		Female		Male		*p*-Value
	*n*	%	*n*	%	*n*	%	
O Rh (−)	179	3.6	141	3.4	38	4.4	0.052 ^1^
O Rh (+)	1492	30.0	1225	29.9	267	30.6	
A Rh (−)	200	4.0	167	4.1	33	3.8	
A Rh (+)	1952	39.3	1611	39.3	341	39.1	
AB Rh (−)	28	0.6	22	0.5	6	0.7	
AB Rh (+)	323	6.5	280	6.8	43	4.9	
B Rh (−)	74	1.5	69	1.7	5	0.6	
B Rh (+)	721	14.5	581	14.2	140	16.0	
Total	4969	100.0	4096	100.0	873	100.0	

^1^ Chi-square test.

**Table 3:** Eight-category ABO/Rh(D) distribution: observed vs. expected (Antalya reference).

Blood Group	Observed (*n*)	Observed (%)	95% CI (%)	Expected (*n*)	Expected (%)	O-E	Standardized Residuals
O Rh (−)	179	3.60	3.12–4.16	171.43	3.45	+7.57	+0.58
O Rh (+)	1492	30.03	28.77–31.32	1561.76	31.43	−69.76	−1.77
A Rh (−)	200	4.03	3.51–4.61	199.75	4.02	+0.25	+0.02
A Rh (+)	1952	39.29	37.93–40.65	1937.91	39.00	+14.09	+0.32
AB Rh (−)	28	0.56	0.39–0.81	39.26	0.79	−11.26	−1.80
AB Rh (+)	323	6.50	5.85–7.22	283.73	5.71	+39.27	+2.33
B Rh (−)	74	1.49	1.19–1.87	63.60	1.28	+10.40	+1.30
B Rh (+)	721	14.51	13.56–15.52	711.56	14.32	+9.44	+0.35
Total	4969	100.00		4969.00	100.00	—	

Chi-square goodness-of-fit (8-category): χ^2^ (7) = 14.04, *p* = 0.050. O-E: Observed-Expected. CI: Confidence Interval.

**Table 4:** ABO-only distribution: observed vs. expected (Antalya reference).

ABO Group	Observed (*n*)	Observed (%)	95% CI (%)	Expected (*n*)	Expected (%)	O-E	Standardized Residuals
A	2152	43.31	41.94–44.69	2137.66	43.02	+14.34	+0.31
O	1671	33.63	32.33–34.95	1733.19	34.88	−62.19	−1.49
B	795	16.00	15.01–17.04	775.16	15.60	+19.84	+0.71
AB	351	7.06	6.38–7.81	322.99	6.50	+28.01	+1.56
Total	4969	100.00		4969.00	100.00	—	

Chi-square goodness-of-fit (ABO-only): χ^2^ (3) = 5.26, *p* = 0.153. O-E: Observed-Expected. CI: Confidence Interval.

**Table 5:** Rh(D)-only distribution: observed vs. expected (Antalya reference).

Rh Group	Observed (*n*)	Observed (%)	95% CI (%)	Expected (*n*)	Expected (%)	O-E	Standardized Residuals
Rh (+)	4488	90.32	89.47–91.11	4494.96	90.46	−6.96	−0.10
Rh (−)	481	9.68	8.89–10.53	474.04	9.54	+6.96	+0.32
Total	4969	100.00		4969.00	100.00	—	

Chi-square goodness-of-fit (Rh-only): χ^2^ (1) = 0.11, *p* = 0.737. O-E: Observed-Expected. CI: Confidence Interval.

**Table 6:** Summary of published studies evaluating the ABO blood group and osteoarthritis.

Author (Year)	Country	OA Site	Study Design/ #Case vs. Controls	Main Findings
Lourie (1983) [23]	UK	Primary hip OA (THR patients)	Retrospective case–control study (341 vs. 7072)	ABO distribution showed a modest difference; overall, the 4-group comparison was non-significant, but group O was less frequent than non-O among OA patients.
Li et al. (2019) [19]	China	Primary knee OA	Retrospective case-control study (1126 vs. 3030)	AB blood group was associated with increased risk of primary knee OA (reported as an independent risk factor), and synovial LeY antigen expression was linked to disease severity.
Yaradilmis et al. (2021) [25]	Türkiye	Primary knee OA (TKA patients)	Retrospective case-control study (658 vs. 2322)	ABO distribution differed between groups; the frequency of A blood type was higher in OA patients, and A blood type was reported as an independent risk factor for primary knee OA.
Shaikh et al. (2024) [24]	Pakistan	Primary knee OA	Comparative observational/case–control-like hospital-based study (190 vs. 380)	A blood group (especially A+) was more frequent among OA cases; the authors reported a significant association of ABO type with knee OA and severity.
Nwachukwu et al. (2024) [26]	Nigeria	Severe knee OA	Observational study (no control arm, *n*: 116)	The authors reported no significant link between the ABO blood group and the development of severe OA in their cohort.
Current Study (2026)	Türkiye	Primary knee OA (primary TKA patients)	Retrospective distributional comparison using an external donor-based regional reference population (4969 vs. 10,867)	Compared with the regional reference distribution, the 8-category ABO/Rh(D) distribution showed a borderline difference (*p* = 0.050), while ABO-only and Rh(D)-only distributions were not significantly different.

Abbreviations: OA: Osteoarthritis, THR: Total hip replacement, TKA: Total knee arthroplasty.

## Data Availability

The datasets are not publicly available. The de-identified data are available upon request to the corresponding author due to privacy, ethical, and legal restrictions protecting patient confidentiality.

## Abbreviations

The following abbreviations are used in this manuscript:

OA	Osteoarthritis
TKA	Total knee arthroplasty
ABO	ABO blood group system
Rh(D)	Rhesus D blood group antigen
TNF-α	Tumor necrosis factor-alpha
ICAM-1	Intercellular adhesion molecule-1
LeY	Lewis Y antigen
SD	Standard deviation
*n*	Number
O−E	Observed minus expected
THR	Total hip replacement
